# Prevalence, correlates, and prospective predictors of non-suicidal self-injury among New Zealand adolescents: cross-sectional and longitudinal survey data

**DOI:** 10.1186/s13034-015-0055-6

**Published:** 2015-07-08

**Authors:** Jessica Anne Garisch, Marc Stewart Wilson

**Affiliations:** School of Psychology, Victoria University of Wellington, P.O. Box 600 Kelburn Parade, Wellington, New Zealand

## Abstract

Non-suicidal self-injury (NSSI) is common among adolescents and linked to many maladaptive outcomes. This study aimed to assess the prevalence and correlates of NSSI among a community sample of New Zealand adolescents. A self-report questionnaire was administered to adolescents at time 1 (*N* = 1162, mean age = 16.35), and approximately five months later (time 2, *N* = 830, mean age = 16.49). Prevalence and bivariate correlations were assessed at both time points, and cross-lag correlations using matched data (*N* = 495, mean age = 16.23). Lifetime history of NSSI was 48.7 % (females 49.4 %, males 48 %). Consistent with previous international research, NSSI was associated with higher Alexithymia, depression, anxiety, bullying, impulsivity, substance abuse, abuse history and sexuality concerns and lower mindfulness, resilience and self-esteem. Cross-lag correlations suggested NSSI is directly (perhaps causally) related to psychological vulnerability in various domains (e.g., increased depression and lower self-esteem), while bullying may be more distal to NSSI, rather than a proximal predictor.

Non-Suicidal Self-Injury (NSSI) is defined here as the intentional, culturally unacceptable, self-performed, immediate and direct destruction of bodily tissue that is of low-lethality and absent of overdose, self-poisoning and suicidal intent. Suicidal self-injury is viewed as qualitatively different to NSSI (e.g. [4, 61]). Self-reported lifetime history of NSSI among adolescents ranges from between 7 and 66 %, depending on the definition and self-report measure used (e.g. [3, 20, 33, 34, 39, 42]). NSSI is associated with a variety of comorbid difficulties that suggest underlying emotional and/or social distress [48]. For this reason, it is important for researchers and clinicians to disentangle which psychological variables co-occur with NSSI, and which are significant risk and protective factors. In spite of a growing body of research regarding the correlates of NSSI, there is a need for longitudinal studies to assist in identifying potentially causal factors (see, for example, [70]).

This study investigates prevalence, correlates, and prospective predictors of NSSI among New Zealand adolescents. There is currently no large-scale research involving New Zealand adolescents, assessing the prevalence of NSSI using a multi-item measure of self-injury. Previous New Zealand research has either involved adults (e.g. [40, 60]), been based on hospital admissions (e.g. [8]) or clinical populations (e.g. [16]), or does not distinguish between behaviours with or without suicidal intent (e.g. [32]). Where large-scale community samples of adolescents have been used, self-injury is assessed using only one or two items (e.g. [8, 35]) that do not allow differentiation between NSSI and deliberate self-harm (DSH; which does not preclude suicidal intent), and are cross-sectional. As a result, there is currently no information about the prevalence of NSSI in New Zealand.

Similar methodological issues beset international studies to the issues described above (e.g. not excluding behaviours with suicidal intent, using single item measures; e.g. [34, 35, 64]). A review of the international literature on longitudinal studies of NSSI and DSH suggests wide variation in the measurement of self-injurious behaviour, the length of follow-up, and the types of predictors that various researchers include (see [48]). Plener et al’s [48] review indicates that past NSSI is one of the strongest predictors of future NSSI behaviour, and other consistently reported predictors include depressive symptoms, female gender, suicidality and psychological distress. However, understanding of the longitudinal development and cessation of NSSI remains a new area of research with inconsistent findings and methods across samples.

Tuisku et al. [68] report a longitudinal study of Finish adolescent outpatients, indicating that past NSSI was the only prospective predictor of NSSI at 8-year follow-up (perceived social support, anxiety and depressive symptoms were not predictive). Stallard et al. [64] followed community adolescents in England over a 6 month period. Symptoms of low mood and insecure peer attachments were predictive of self-harm for both males and females, whilst alcohol use was not predictive for either sex. Cannabis use was predictive of self-harm ideation for males, and self-harm behaviour for females. Use of street drugs and being bullied was predictive of self-harm for males only. Marshall et al. [34] conducted a three-wave longitudinal study investigating the link between depression and NSSI among community adolescent sample. Although depression at T1 predicted NSSI at T2, T2 depression did not predict T3 NSSI, suggesting that adolescents who self-injure may become more heterogeneous with age.

We are aware of only one published longitudinal study conducted in New Zealand investigating self-injurious behaviour. Nada-Raja and colleagues [41, 42], as part of the Dunedin Multidisciplinary Health and Development Study [58], report different prospective predictors depending on sex; for women history of assault victimisation, posttraumatic stress disorder symptoms and anxiety disorders was predictive of self-harm at age 26 whilst for men this was only true for anxiety and depressive symptoms.

Here, we investigate prevalence of NSSI in a large community sample with a multi-item instrument for self-reported NSSI behaviour, over two time points. Such a design allows us to make (after accounting for stability of constructs over time, and their cross-sectional relationships) inferences concerning prospective predictors.

The predictor variables included in this study are not an exhaustive list of correlates of NSSI, but include those potential risk and protective factors most strongly related to NSSI in international literature. This study investigates psychological correlates of NSSI including Alexithymia (the ability to understand and communicate emotion, [57]), self-esteem, adaptive use of emotion, depression, anxiety, resilience, mindfulness, impulsivity, and sexuality concerns, as well as victimisation (i.e. abuse and bullying history), and behavioural correlates (i.e. substance use).

NSSI has been shown to be consistently associated with higher scores on measures of depression and anxiety (e.g., [9, 23, 38, 55, 34, 64, 71]). Research indicates that depression may be causally related to NSSI [71]. These negative affective states reduce during, and especially after, an episode of NSSI, accompanied by a sense of relief [30, 43]. Nixon et al. [43] suggest that NSSI may be a self-medicating mechanism for depression, especially considering the affect-modulating and addictive qualities of NSSI endorsed by their sample.

NSSI has been linked to factors indicative of poor self-perception and integration of identity [5], including low self-esteem [11, 22, 30, 33, 35]. This may be especially pertinent for youth, as a primary developmental task of adolescence is identity formation and the development of close extra-familial interpersonal relationships [65]. A related adolescent task is the development of sexuality, and same-sex attraction may be a risk factor for self-injuring behaviour among youth [32, 60].

NSSI is associated with low mindfulness [33], impulsivity (see [71], for indication of a longitudinal relationship), poor emotional awareness, low cognitive reappraisal and emotional repression [1], and lower resilience [13] all internal resources for self-management. NSSI is associated with poor awareness of internal psychological processes, with research linking NSSI to Alexithymia and poor emotional regulation and intelligence [1, 12, 18, 20, 30, 46]. We use the term ‘adaptive use of emotions’ to represent the ability to manage and understand emotions (see [26], for further discussion). Fostering emotional understanding and tolerance of emotional distress is a common part of therapeutic intervention for NSSI [36].

All types of childhood abuse and trauma have been linked to NSSI [23, 41, 63]. NSSI may provide an escape from trauma symptoms, with NSSI being negatively reinforced through the removal of unwanted symptoms (e.g. intrusive memories, dissociation), leading to the potential maintenance of NSSI over time (see [63] for a review). Research suggests NSSI is significantly more prevalent among bullied adolescents [53, 7, 18, 23], and a history of bullying is longitudinally predictive of NSSI [15, 31]. NSSI and being bullied both co-vary with negative psychological outcomes [2, 49, 30, 38, 7, 11]. An individual who engages in NSSI may also be an easy target for a bully due to low self-esteem and poor emotion regulation (i.e. easily intimidated and emotionally responsive). Additionally, self-injuring youth may actively seek out persecution from others as an extension of their self-injury (similar to how some researchers consider remaining in an abusive relationship to be NSSI; [20]).

NSSI has been linked to alcohol, tobacco, and illegal drug use [23], though the causal role remains unclear (e.g. see Stallard et al. [64] and [68]). Evans et al. [12] found that self-harming adolescents were more likely to have an alcoholic drink when angry or upset than non-NSSI adolescents. Both NSSI and substance abuse reflect an avoidant coping style; neither resolves the individual’s underlying issue(s) but may be utilised for short-term relief. Desire for short-term relief is associated with impulsivity, another correlate of NSSI [23].

While there is a growing literature that attests to the potential roles of the constructs described above, the evidence on their potentially causal roles is mixed and at times even contradictory. For example, there is research to indicate that depression might predispose an individual to NSSI (e.g. [64]), be a consequence of NSSI (e.g. [71]), or that the two co-occur/run alongside each other but do not have a causal relationship. Alternatively, depression and NSSI may reciprocally influence each other. This study set out to investigate prevalence, cross-sectional and cross-lagged correlates of NSSI among New Zealand adolescents. A large sample of New Zealand adolescents has not been assessed using a multi-item measure of NSSI, with analysis of cross-sectional and cross-lag correlations to allow for some investigation of prospective relationships. While this is a novel study in the New Zealand context, it is also one of only a few studies to have investigated NSSI and its correlates over time internationally among community adolescents (see [48] for a review). Additionally, studies typically show little evidence that many of the constructs routinely correlated with self-injury are actually causally implicated in its development or maintenance (e.g. [68]).

To address our aim, a self-report survey was administered across two time points (T1 and T2) approximately 5 months apart. It was hypothesised we would identify prevalence rates falling within the 7-66 % band previously identified. Given the use of a multi-item measure, and the consistent finding that such measures typically result in higher prevalence rates, we anticipated that the figure would be in the top half of this range. Additionally, we anticipated that all predictor variables assessed in the survey (i.e. depression, anxiety, self-esteem, Alexithymia, resilience, mindfulness, adaptive use of emotions, bullying, abuse history, substance abuse, sexuality concerns; the literature linking these variables to NSSI is described above) would be significantly correlated with NSSI. The cross-lag correlations are exploratory, particularly given the contradictory findings in previous literature; for that reason we make no directional predictions at this point.

## Method

### Participants

Participants were students at capital city-area secondary schools. All 31 secondary schools in the Wellington region were approached, and ten schools agreed to participate, including public (state-funded) and private schools, and mixed-sex as well as single-sex schools. School deciles ranged from 3 to 10 (mean = 7.6, SD = 2.54) where decile indicates the extent a school draws its student population from low socioeconomic communities (from 1 to 10, where 10 means few students from low socioeconomic status backgrounds). Students in years 12 and 13 (aged 16 and over) were invited to participate (it is legal convention in New Zealand that young people aged over 16 may consent on their own behalf without explicit parental consent to opt-in). The average participation rate was 60 % (ranging from 51 % to 84 %; slightly better than the average 56 % response rate reported by [45], in a review of survey response rates).

Time 1: Participants were 1162 (43 % female) secondary school students with an average age of 16.35 years (S.D = .62). 71.1 % self-identified as Pākehā/NZ European, 8.8 % as Māori (indigenous New Zealanders), 20.1 % as 'other'.

Time 2: There were 830 (47 % female,) participants, mean age of 16.49 years (SD = .71). Broken down by ethnicity, 66.9 % identified as Pākehā/New Zealand European, 8.2 % as Māori, and 21.7 % as 'other'.

Participants for matched dataset: 495 (48 % female, mean age = 16.23, SD = .56) of the 1162 that completed T1 were matched by identifier to T2 data. 74.6 % identified themselves as Pākehā, 8.9 % as Māori, and 16.5 % as 'other'. This ethnic break-down is similar to that found for the entire T1 sample.

Comparison of the sample with government statistics (Ministry of Education [37]) for the Wellington region indicated that the samples were representative of socioeconomic status and student sex, but that the samples were over-represented by Pākehā/NZ European and under-represented by Māori students. Several factors account for the high attrition. Fifty-four participants either did not give a unique identifier or gave an incomplete identifier at T1. Also elements of the unique identifier may have changed for participants over the time period (e.g. phone number), or participants may have changed schools (especially in one school where participation spanned two academic years), or not been present at the second administration of the survey. As participation was voluntary, some students may have chosen not to take part in the survey a second time or made an active choice not to facilitate data matching.

### Measures

All measures were self-report, and chosen for sound psychometric properties and brevity. Measures were identical at T1 and T2 survey distribution, except the measure of NSSI, where at T1 lifetime NSSI was assessed, and at T2 NSSI since the first survey distribution (i.e. past 3–8 months) was assessed.

*Non-suicidal self-injury* was assessed using the Deliberate Self-Harm Inventory – Short form (DSHI-s; [33]) that asks about multiple forms of NSSI behaviour. Multi-item measures increase reliability and ensure a wider range of NSSI is identified [33]. DSHI-s behaviours are low-lethality, behaviourally precluding suicidal intent, and completed on a 5-point scale from “Never” to “Many times” engaging in the specified NSSI behaviour.

*Depression* and *anxiety* were measured using the 20-item Self-rating Depression Scale (SDS; [72]) and 20-item Self-rating Anxiety Scale (SAS; [73, 74]). Participants rated items on a 4-point Likert scale (1 ‘none of the time’ to 4 ‘most of the time’), according to how they feel at the time of participation. Both scales have good psychometric properties [73, 74, 28].

*Self-esteem* was measured using Rosenberg’s 10-item Self-esteem Scale (RSE; [50]), developed for use with adolescents, and with good validity and reliability [50, 52]. Each item is assessed on a 4-point Likert scale from “strongly agree” to “strongly disagree”.

*Alexithymia* was assessed using the 20-item Toronto Alexithymia Scale (TAS-20; [66]) using a 7-item Likert scale (1 ‘strongly disagree’ to 7 ‘strongly agree’). The TAS-20 shows satisfactory internal reliability (α = .78) and we have previously used this with secondary school students [18].

*Adaptive use of emotions* was assessed with the 33-item Schutte [56], developed for use with adolescent community populations, and is reliable (α = .89; [54]), and rated on a 1 (‘Very seldom’) to 5 (‘Very often’) scale.

*Resilience* was measured using the reliable 15-item (1 ‘strongly disagree’ to 7 ‘strongly agree’) scale developed by Wagnild and Young [69]; α = .91.

*Mindfulness* was assessed using the 12-item Cognitive and Affective Mindfulness Scale – Revised (CAMS-R; [18]; 1 = 'rarely/not at all, 4 = 'almost always'). The scale is appropriate for reliable use with adolescents [14].

*Sexuality Concerns* were assessed by the single item “Have you ever worried about issues around sexuality (e.g., being straight, gay, etc.)?”; used previously [18]. There were four possible responses; “no”, “yes, once”, “yes, a lot”, and “decline to say”.

*Impulsivity* was measured using the 30-item Barratt Impulsivity Scale (BIS II, [47]; from 1 'rarely/never' to 4 'almost always/always'). The BIS II is reliable and widely used (α = .83; for a review see [62]).

*Bullying* was assessed using questions from Section D of the Peer Relations Questionnaire [49], asking recency of bullying and frequency of six different types of bullying (rated from 1 'never' to 3 “often”). We added an item on electronic bullying as this has been linked to NSSI [18].

*Abuse history*^1^ was assessed with a 2-item screening instrument [67]. The items are “When I was growing up, people in my family hit me so hard that it left me with bruises or marks”, and “When I was growing up, someone tried to touch me in a sexual way or tried to make me touch them”. These items were rated on a 5-point scale from1 (“never”) to 5 (“very often”) [67].

*Substance use* was assessed by asking participants if they had used cigarattes, alcohol "to excess", "(legal) party pills", "illegal drugs (e.g., Cannabis, etc.)”, “Have you ever smoked a cigarette?” (response options were 'No', 'Yes, once', and 'Yes, more than once').

The survey began with an information sheet, and ended with a (removable) contact sheet.

### Procedure

Typical process involved speaking to students about the study 1–2 weeks before survey administration. Depending on the preference of school administration participation occurred during class or form room period, or in large groups in the school hall, under supervision of their teacher and/or the researcher. Before survey administration students were reminded that participation was voluntary and anonymous, and that completion and return of the survey indicated consent for use of their anonymous responses. In all but one school (20 min), participants were given approximately 40–50 min to complete the survey. Debriefing sheets were later put up on school notice boards. The modal time between administrations was 5 months, and was based on when schools were willing to have the survey disrupt curriculum work. In order to match data, each participant was invited to supply a unique identifier of their choice (for use in matching surveys). Ethical approval for this study was provided by a University delegated ethics committee representing the National Health and Disability Ethics Committee.

### Statistical methods

Internal reliabilities and test-retest correlations were calculated for all multi-item scales. Pearson's correlations were conducted to assess the relationships between predictor variables an NSSI at T1, at T2, and predicting T2 NSSI from T1 variables.

Having data across T1 and T2 allowed cross-lag panel correlations to be conducted to assess the relationships between each predictor variable and NSSI across time. A cross-lag correlation involves two constructs measured at T1 (X_1_, and Y_1_) and again at T2 (*X*_2_ and Y_2)_, and assesses the strength of the relationship between the two constructs across time (X_1_ with Y_2_, Y_1_ with *X*_2_), while controlling for measurement error and spuriousness (e.g., by partialling out Y_1_ from the X_1_ and Y_2_ cross lag correlation; [27]). Cross-lag correlations were performed using AMOS [version 20] using the T1 and T2 matched sample data for each predictor variable and NSSI, with the exception of that between abuse history and NSSI, due to the historical nature of the questions and because several participants did not complete the abuse items at T1. Error terms were modelled in the analyses, but are not presented.

## Results

All measures with at least three items demonstrated acceptable internal reliability (α's > .70) while the two-item scale for abuse history (r's = .32 and .38, p's < .001) showed satisfactory inter-item correlations at both T1 and T2. With the exception of bullying (test-retest r = .37, *p* < .001) and Schutte scores (test-retest r = .49, *p* < .001) all scales achieved test-retest correlations of at least .52 (*p* < .001).

Table 1 presents prevalence rates for the different types of NSSI at Time 1. The most common was sticking sharp objects into the skin, and the least common breaking bones. T1 prevalence for lifetime history of NSSI at least once was 48.7 % (females 49.4 %, males 48 %); There was no significant difference between males (mean = 1.29, SD = .51) and females (mean = 1.31, SD = .49) for DSHI-s scores at T1, t(1137) = .42, *p* = .67. 12.16 % of those reporting NSSI history indicated most recent episode within the last week, 13.15 % within the last month, 28.29 % within the last year, and 46.40 % as over a year ago. Prevalence rates of NSSI during the follow-up period for the T2 dataset was 34.48 %.

**Table 1:** Lifetime history of different types of NSSI in T1 sample

Type of NSSI	Ever engaged in (%)	Thought about (%)	Once (%)	More than once (%)	Many times (%)
Stuck sharp objects into the skin e.g., pins, needles, staples.	20.19	1.98	8.28	8.37	3.54
Carved words/designs into skin	17.92	3.45	9.56	6.03	2.23
Scratched skin until bled/scarred	15.70	1.56	8.63	3.97	3.02
Cut	14.22	6.90	5.26	5.26	3.71
Punched oneself	14.04	2.07	7.92	4.65	1.46
Banged head	13.82	3.20	8.03	3.37	2.42
Burned with cigarette/lighter	13.52	2.41	7.24	4.22	2.07
Prevented wounds from healing	13.40	2.59	5.27	4.67	3.46
Bit the skin until broken	8.89	1.56	5.09	2.68	1.12
Rubbed sandpaper on the skin	7.92	.34	5.08	1.55	1.29
Dripped acid onto the skin	4.93	.78	3.37	.61	.95
Rubbed glass into the skin	2.84	.95	1.21	1.03	.60
Scrubbed bleach/oven cleaner into the skin	2.24	.69	1.29	.60	.34
Broken bones	1.81	1.38	.95	.52	.34

Table 2 presents cross-sectional correlations between NSSI and the various predictor variables at T1, and at T2, and the correlations between T1 predictor variables and T2 NSSI (i.e. NSSI during the period between survey administrations). After adjustments for multiple tests, all T1 and T2 variables were significantly associated with NSSI at the respective time points, and all but three T1 variables (Schutte adaptive use of emotions, Impulsivity, and bullying) were significant predictors of T2 NSSI.

**Table 2:** Cross-sectional correlations between predictor variable scores and NSSII-s scores at T1 and T2, and correlations between T1 predictor variables and T2 NSSI (i.e. NSSI over 3–8 month period

	T1 predictors with T1 (lifetime) NSSI	T2 predictors with T2 (past 3-8mth) NSSI	T1 predictors with T2 NSSI
Alexithymia (TAS-20)	.37	.33	.18
Self-Esteem (RSE)	-.34	-.41	-.25
Adaptive use of emotions (Schutte)	-.15	-.19	-.10 ns
Anxiety (SAS)	.35	.41	.19
Depression (SDS)	.38	.40	.28
Resilience	-.34	-.33	-.27
Mindfulness (CAMS-R)	-.28	-.26	-.19
Impulsivity (BIS II)	.24	.20	.14+
Bullying (PRQ)	.31	.21	.12 ns
Sexuality concerns	.23	.20	.15
Substance abuse	.32	.25	.19
Abuse history	.39	.35	.24

Note: To address the issue of inflated family-wise error associated with multiple tests, a Bonferroni correction was applied. All correlations significant unless suffixed + (adjusted *p* = <.10) or ns (adjusted *p* non-significant)

### Cross-lag correlations

**Fig. 1:**
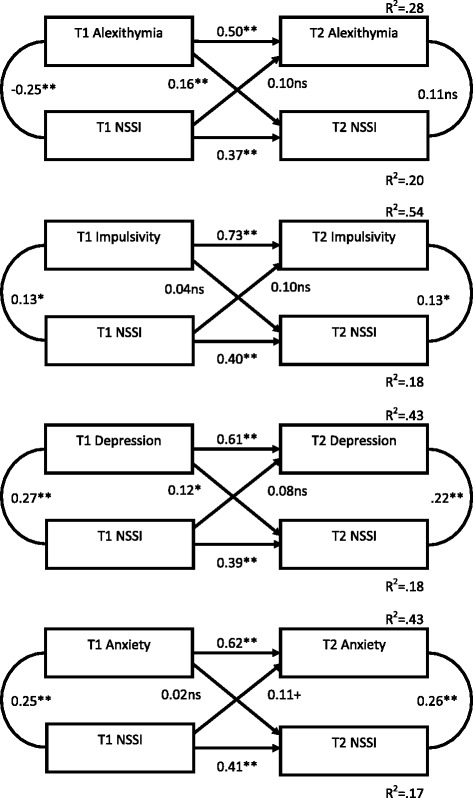
Cross-lagged panel correlations of non-suicidal self-injury and ‘risk’ factors across time 1 and time 2

**Fig. 2:**
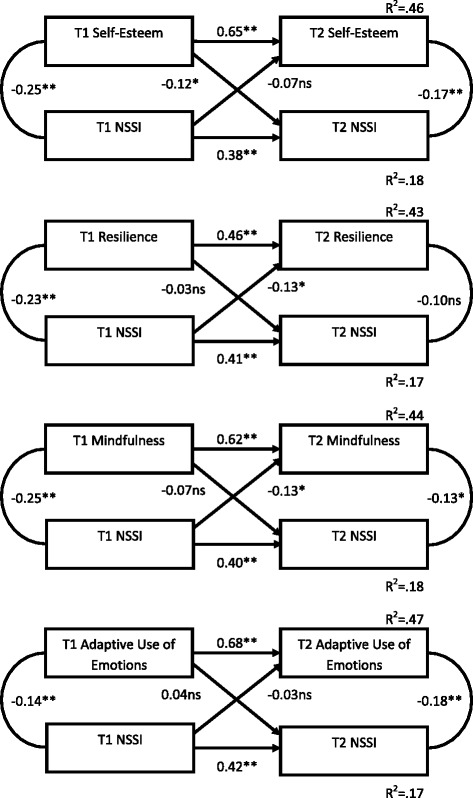
Cross-lagged panel correlations of non-suicidal self-injury and ‘protective’ factors across time 1 and time 2

**Fig. 3:**
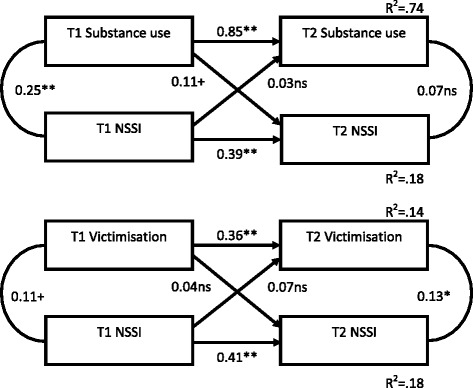
Cross-lagged panel correlations of non-suicidal self-injury and behavioural/contextual factors across time 1 and time 2

Figure 1 represents the cross-lagged panel correlations of NSSI and risk factors, while Figs. 2 and 3 show the results for NSSI and protective factors and behavioural/contextual factors respectively (standardised coefficients are shown).

In all cases, the stability coefficients for NSSI from T1 to T2 were relatively low, indicating that NSSI was more unstable than many of the other constructs assessed. There were no significant cross-lag relationships between NSSI and either bullying, substance use, impulsivity, anxiety, and adaptive use of emotions. However, the 'risk' factors of depression and Alexithymia at T1 were significantly predictive of NSSI at T2 suggesting they may be prospective predictors of NSSI (while not in turn being affected by NSSI). Additionally, the potentially 'protective' factors of self-esteem, mindfulness and resilience NSSI evidenced significant cross-lags - better self-esteem, mindfulness and resilience at T1 predicted lower NSSI engagement at T2.

Thus NSSI appears, in some cases, to be exacerbated by the presence of some (but not all) risk factors, and ameliorated by others.

## Discussion

Prevalence rates for lifetime history of NSSI in this study were higher than those reported in many previous international studies of youth NSSI - almost 50 %. However, previous research using the DSHI-s has found high (and indeed higher) lifetime prevalence of NSSI among adolescents (e.g. 41.5 % in the past six months: [3]; 65.9 %: [33]). This is attributed to the use of a range of items; the majority of previous research has asked about a limited range of NSSI (e.g., cutting) and consequently may have missed identifying youth who self-injure using different methods (e.g., prevalence rates of 7.2 % to 14.8 % for NSSI among adolescent samples using single- or two-item measures: e.g., [9, 51]).

No sex difference in overall prevalence was found, contradicting the stereotype that NSSI is more common among females than males [10]. Again, this may be due to the assessment of a limited range of behaviours (e.g., [23]). Similarly, shorter measures typically assessing for prototypical self-injuring behaviours (e.g. cutting; which females in this sample self-reported more) may under-estimate male NSSI. However, overdose is a more common form of self-harm among females [23], and was excluded in the definition (and measurement) of NSSI in this study.

The cross-sectional results support those found in the literature internationally, with all predictor variables significantly correlated with NSSI in this large New Zealand adolescent sample. Using cross-lag panel analyses, several of these correlates became non-significant predictors across time; for example, adaptive use of emotions and bullying. This is potentially consistent with models of NSSI where psychological or self-regulatory factors (e.g., depression, self-esteem) are seen as proximal and central to self-injury, whereas social or environmental factors (e.g., bullying) are seen as more distal factors (e.g., Experiential Avoidance Model or EAM, [6]; a diathesis stress model of NSSI; [44]).

The results of the cross-lag correlations suggest that there is a consistent pattern whereby engaging in NSSI is associated with poorer subsequent psychological functioning (i.e. greater, but not significantly so, endorsement of depressive symptoms, lower self-esteem, resilience, and mindfulness). NSSI may cause anxiety relating to scars and discovery (known concerns among youth who self-injure; [24]), and to a sense of loss of control as it becomes more ingrained and relied upon to cope with everyday distress. Perhaps engaging in NSSI for an extended period lowers personal coping resources (resilience) as the behaviour becomes habitual (see addictive qualities of NSSI; [43]). Alexithymia proved an important construct and, in combination with problematic mood, likely creates vulnerability to using NSSI as an escape from strong emotional experience, or as an alternative form of emotional expression. Indeed, the EAM [6] suggests that deficits in emotion regulation skills play an important role in inappropriate responses to environmental stresses.

Numerous studies correlate NSSI with depression in youth (e.g., [34, 64, 71]), and narrative accounts suggest NSSI often occurs in the context of depression [59]. Longitudinal research is mixed on whether depression is predictive of NSSI over time, with some findings supporting a causal relationship (e.g. [21, 64]), others suggesting NSSI increases depressive symptoms but not the reverse (e.g. [71]), and still others pointing to a complex scenario whereby the heterogeneity of youth who engage in NSSI makes this relationship very difficult to disentangle and the strength of various predictors, including depression, may change over development, or these variables may co-occur but not be causal to NSSI (e.g. [34]). The recent review by Plener et al. [48] indicates that depressive symptoms are among the more consistent predictors of NSSI, and our results further corroborate this finding.

Positive self-esteem appears to buffer against NSSI, consistent with a body of research identifying self-image as being vital in NSSI and coping generally. Additionally, Self-esteem may decrease post-NSSI due to internalising negative stigma (e.g., NSSI as attention seeking and manipulative; [17]). The relatively immediate relief or distraction from emotional or internal experience that NSSI offers (see data on personal accounts; [43]) is incompatible with a mindful stance of non-judgement, acceptance, and awareness of emotional experience [19]. Over time NSSI may lead to intolerance of emotion and internal distress, or internal distress may be more quickly rejected and trigger self-injury as an escape mechanism, at the expense of being mindful of emotions. This is consistent with research indicating that emotional suppression is associated with the continuation of NSSI over time [1]. Along with the significant buffering effect of mindfulness and resilience, there is reason to think that interventions focusing on self-esteem, resilience, and mindfulness may be useful.

The results from the cross-lag analyses suggest low mood, substance abuse, low self-esteem and Alexithymia are proximal predictors of NSSI, and engagement in NSSI reduces resources for ongoing self-management (e.g. lower resilience, mindfulness, and self-esteem/sense of self-efficacy and increased impulsivity). These findings (the first longitudinal study of Alexithymia and NSSI) suggest an underlying avoidant coping style (e.g. to use substances to self-medicate; to seek immediate relief for negative emotions), in the presence of a weak repertoire of emotional skills, which is reinforced with continued NSSI. This indicates a downward spiral of increasing reliance on NSSI to manage internal distress, and suggests early intervention may be useful in preventing the ongoing damage of internal self-management were NSSI to continue. Again, this is congruent with the EAM [6]. According to the EAM, NSSI is utilised to regulate negative emotion, whilst the after-effects of NSSI (e.g. shame, guilt) fuel further negative internal experience and reduced ability to cope over time, and NSSI re-occurs.

The longitudinal results require replication. Previous research assessing the relationship between these variable and NSSI across time using an adolescent sample have inconsistent findings and/or do not assess NSSI appropriately, and there has been little longitudinal research on NSSI in New Zealand and internationally (e.g., see [48] for a review). The field would also benefit from complex, empirically based, models of NSSI incorporating multiple predictors. In general, existing empirical models based on longitudinal data include only a few predictor variables (e.g. cross-lag models with two independent variables; [71]). Until this is done, there continues to be the risk that prospective relationships as identified here may be the product of a third, omitted, variable. The predictors of NSSI do not occur in a vacuum, and it is important to understand how the various predictors fit together to create vulnerability to NSSI.

The study had several limitations. Given that the research is entirely based on self-report, and around 60 % of T2 surveys could be matched (in spite of the fact that more than 80 % of T1 participants completed both surveys, meaning that a significant group of participants either accidentally or wilfully provided inconsistent unique identifers), we have concerns around the potential for bias. For example, due to limited/censored disclosure resulting from the stigma of NSSI [17], and potentially exacerbated by sensitivities around abuse and sexuality [25, 29]. The anonymity of the surveys was designed to encourage open and honest disclosure; however the fact that the youth surveys were completed in groups may have led some to be concerned that their responses were observable by peers. There was some amount of variation in the period between T1 and T2 that, had we a larger sample to counter the low power of cross-lagged correlation analyses [27], would have been best addressed by statistically taking this into account.

Recent research indicates that multi-wave longitudinal studies are needed, with separate analyses by cluster groupings of adolescents who engage in NSSI (e.g. chronic NSSI; clustered types of NSSI) and sex, to account for the variance in predictors due to the heterogeneity in the behaviour and developmental stages (e.g. see [3, 34]). Past studies indicate that a two-wave design does not necessarily demonstrate the true relationship between variables (e.g. [34]). Future research will need to be multi-wave, and separate samples by sex and into clusters based on frequency and method of NSSI (e.g. see [3]) to fully appreciate the longitudinal relationships between risk and protective factors and NSSI.

Overall this study, the first of its scope and nature in New Zealand, suggests that NSSI is highly prevalent among New Zealand secondary school students, both for males and females, with almost half of the participants reporting a lifetime history of NSSI at least once. Analyses indicate that NSSI co-occurs with various indicators of psychological (e.g., depression, anxiety, low self-esteem and poor mindfulness) and social (e.g., being bullied) distress, and that NSSI leads to poorer psychological functioning. Importantly, cross-lagged analyses suggest that at least some of these robust correlates co-occur but may not prospectively influence NSSI. Over time NSSI may lead to decreased internal regulation and self-management (i.e. increased anxiety and impulsivity). It will be important to intervene early to support young people who self-injure to help prevent a downward spiral of engaging in NSSI to manage internal distress, and the analyses presented here suggest successful interventions may be those that promote mindfulness, resilience and self-esteem.

## Abbreviations

BIS II: Barratt impulsivity scale
CAMS-R: Cognitive and affective mindfulness scale – revised
DSH: Deliberate self-harm
DSHI-s: Deliberate self-harm inventory – short form
MOE: Ministry of Education
NSSI: Non-suicidal self-injury
NZ: New Zealand
RSE: Rosenberg’s self-esteem scale
SAS: Self-rating anxiety scale
SDS: Self-rating depression scale
T1: Time 1
T2: Time 2
T3: Time 3
TAS-20: Toronto alexithymia scale

## Footnotes

1 Questions on abuse were only included in later versions of the school survey because the topic of NSSI proved controversial among secondary schools, and adding questions on abuse in addition to the sensitive questions on NSSI, bullying, substance abuse, and sexuality may have discouraged participation. Thus, the first three schools surveyed did not have abuse questions included in T1 surveys for their students. Abuse questions were included in later administrations when no negative feedback from students or guidance counsellors was received. Schools were given the explicit option of excluding the abuse questions; only one chose to do so.
